# Opportunities to advance cervical cancer prevention and care

**DOI:** 10.1016/j.tvr.2024.200292

**Published:** 2024-10-25

**Authors:** Grant Brooke, Sebastian Wendel, Abhineet Banerjee, Nicholas Wallace

**Affiliations:** a Division of Biology, Kansas State University, Manhattan, KS 66506, USA; b Department of Kinesiology, Kansas State University, Manhattan, KS 66506, USA

## Abstract

Cervical cancer (CaCx) is a major public health issue, with over 600,000 women diagnosed annually. CaCx kills someone every 90 s, mostly in low- and middle-income countries. There are effective yet imperfect mechanisms to prevent CaCx. Since human papillomavirus (HPV) infections cause most CaCx, they can be prevented by vaccination. Screening methodologies can identify premalignant lesions and allow interventions before a CaCx develops. However, these tools are less feasible in resource-poor environments. Additionally, current screening modalities cannot triage lesions based on their relative risk of progression, which results in overtreatment. CaCx care relies heavily on genotoxic agents that cause severe side effects. This review discusses ways that recent technological advancements could be leveraged to improve CaCx care and prevention.

## 1 Introduction

According to the World Health Organization (WHO), globally, there are over 600,000 women diagnosed with cervical cancer (CaCx) each year. These malignancies kill someone every 90 s [1]. This tremendous public health burden is also not equitably distributed as the majority of CaCx deaths occur in low- and middle-income countries [1]. Unlike cancer in general that is most often diagnosed in the sixth or seventh decade of life, CaCx is most often diagnosed in women 35–44. Because CaCx frequently occurs in younger, working-age women, who live in countries with developing economies, there is also an outsized economic impact from this disease.

Differences in access to healthcare drive the differential impact of CaCx in high and low resource settings [2]. In high resource settings, premalignant cervical lesions are identified through frequent screening via the Papanicolaou test or Pap test. This screening methodology is performed by collecting cervical cells using a small spatula or brush. These cells then undergo analysis by a pathologist specializing in detection and diagnosis of pre-malignant lesions. Regular screening of women via Pap test or other methodology during routine examination effectively prevents CaCx by allowing premalignant lesions to be treated before transformation. Women in low- and middle-income countries often miss out on this preventative care due to lack of infrastructure [3] and are screened via visual inspection with acetic acid (VIA) [4].

At least 90 % of CaCx are caused by human papillomavirus (HPV) infections [5]. HPV is a large family of DNA viruses with genomes roughly 8000 bps in size [6]. The HPV genome is divided into early (E1, E2, E4, E5, E6, and E7) and late (L1 and L2) genes, based on the relative time these genes are expressed during infections. HPV can be divided into five different genera: Alpha, Beta, Gamma, Mu, and Nu based on the sequence of the L1 region [6]. While members of each genus can cause disease, alpha genus HPVs are the most clinically relevant. Alpha HPVs can infect both mucosal and cutaneous epithelium and are sub-divided into high- (e.g., HPV 16 and 18) and low-risk (e.g., HPV 6 and 11) viruses based on their relative ability to cause cancer. High-risk HPVs cause most CaCx as well as other anogenital and oropharyngeal cancers. Low-risk HPVs cause benign genital warts [[7], [8], [9]], and HPVs 6 and 11 can cause recurrent respiratory papillomatosis [10]. Because this review focuses on CaCx, we will refer to high-risk HPVs more simply as “HPVs” moving forward. However, there are excellent reviews from our group and others that focus on other genera of HPV [11].

Lack of access to school-aged vaccination programs also contributes to the unequal distributions of CaCx incidences worldwide. HPV infections are impressively common throughout the globe, with an annual incidence rate exceeding 10 % of adults [12]. In addition to screening, there are multiple highly effective vaccines that can prevent CaCx by preventing HPV infections. However, as HPV is a sexually transmitted virus, HPV vaccination is most effective at stopping CaCx when given to women prior to sexual debut. The most effective way to provide this immunization at the optimal time is either as vaccination program built into school enrollment or attendance for 9–14-year-olds. There are multiple barriers to implementing these protection efforts, but they are harder to bypass in countries with less well-developed school systems. Additionally, many countries and regions rely on health care facilities, community outreach, and vaccination campaigns to provide childhood vaccinations, necessitating the training of new personnel and the development of new procedures to implement school-based vaccination programs [13]. To make matters worse, the HPV vaccine is relatively expensive even with Gavi-eligible pricing ($2.90 USD per dose), pricing it above vaccination budgets for Ministries of Health [[14], [15], [16]].

In addition to the barriers to optimal CaCx prevention, the high cost of drug discovery and development also negatively impact CaCx care. Estimates of the cost to bring a new drug to market range from over $150 million to as high as $4.5 billion [17]. While this is a substantial barrier for the development of any new cancer therapy, the cost benefit analysis becomes less favorable when considering that CaCx primarily impacts women without fiscal means to pay a high cost for care. As a result, there have been few concerted efforts by pharmaceutical companies to modernize CaCx care by introducing new drugs. Indeed, despite the harsh and often dose-limiting side effects, frontline care for CaCx still relies on cisplatin (and other platinum-based drugs), a drug first approved by the FDA in 1978.

This review discusses the limitations of current CaCx screening and treatment as well as ways that recent technological advances could address these shortcomings.

## 2 Efforts to improve preventative care

This section discusses efforts to prevent CaCx, focuses on preventing HPV infections and premalignant lesion detection and intervention. There is a discussion of current gaps in care and a portion dedicated to identifying ways to address these gaps.

### 2.1 State of cervical cancer prevention

Because most CaCx are caused by a viral infection, prophylactic vaccination is a powerful prevention tool. Multiple HPV vaccines (bi-valent, quadri-valent, and nona-valent) are available, typically administered in 2–3 doses [18]. Because HPV is a sexually transmitted disease, vaccination against HPV is most often given at ages prior to sexual debut, or between 9 and 14 years old [[19], [20], [21]]. The nona-valent HPV vaccine is the only version offered in the US as of 2016 and provides immunity against low-risk HPVs 6 and 11 and high-risk HPVs 16, 18, 31, 33, 45, 52, and 58. Quadri-valent vaccines preceded the nona-valent vaccine as the most common and provided immunity against 6, 11, 16, and 18 [19]. The quadri-valent vaccine has reduced the prevalence of HPV infection in US women ages 14–24 compared to the pre-HPV vaccine era [22]. Interestingly, ongoing work suggests that a single vaccination may reduce the incidence of high-grade premalignant lesions (HSILs) [23]. However, there are an estimated 13 million new HPV infections in the US each year [24].

When primary prevention via vaccination fails, detection and screening mechanisms help identify HPV infections and cervical premalignant lesions. HPV screening is typically done using quantitative reverse transcription polymerase chain reaction to detect HPV E6 and E7 expression or hybridization probes that detect HPV DNA [25,26]. HPV-associated disease is also commonly detected via Pap test screening [25,27]. During a Pap test, exfoliated cells are collected from the external and internal cervical os, fixed, and sent to a pathologist. The pathologist examines these cells for signs of transformation such as irregular cytology or changes at the protein level [28,29]. Combining HPV and Pap testing provides additional sensitivity [26]. Approximately 4 out every 5 US women are up to date on Pap screening, contributing to an estimated 50 % reduction in cervical cancer incidence [30]. However, in resource-poor settings, visual inspection with acetic acid (VIA) is often used instead of Pap testing due to the local availability and stability of acetic acid, low technological and training requirements, and quick results [4]. In VIA, 3–5% acetic acid is applied to the cervix, and after 1 min, the cervix is inspected for acetowhite lesions, which result from acetic acid precipitating abnormal proteins in cancer cells. These results can be interpreted immediately by a trained healthcare worker, and the treatment for the lesions can occur on the same day. While VIA reduces cervical cancer mortality, its ability to prevent cervical cancer is unclear [31]. While VIA may be more sensitive compared to Pap screening (87 % in VIA vs 38 % in Pap testing), potentially detecting more lesions, it generally demonstrates lower specificity (73 % in VIA vs 92 % in Pap testing), which can lead to an increased number of false-positive results [32].

Once detected, HSILs are treated by ablative or excisional care. There are a variety of minimally invasive ablative therapies, including cryotherapy, thermal ablation, laser therapy, focused ultrasound, and photodynamic therapy [33]. Cryotherapy is a cost-effective procedure suited for low-resource settings. It uses refrigerant gases (e.g., nitrogen dioxide or carbon dioxide) to freeze lesions. However, it is limited in both depth and area of treatment. Thermal ablation works by heating HSIL tissue to 100–120 °C. Thermal ablation has similar strengths to cryotherapy but includes the risk of cervical stenosis as a notable disadvantage. Laser therapy uses high-precision carbon dioxide lasers to vaporize lesions, with minimal damage to surrounding tissues. It is highly effective but costs more than other ablative methods and has similar common negative impacts. Focused ultrasound has fewer deleterious side effects than most ablative therapies, but it has poorly defined tissue destruction boundaries and limited effectiveness data. Photodynamic therapy has a high efficacy rate and is suitable for fertility preservation, but it is limited by the depth of treatment and requires the administration of a photosensitizer drug.

Excisional approaches include loop electrosurgical excision procedure (LEEP), laser conization, and cold knife conization. Each approach has a high cure rate [[34], [35], [36], [37]]. LEEP uses a fine wire loop with an electrical current to excise lesions, while both laser and cold knife conization surgically remove a cone-shaped section of the cervix [34]. LEEP combined with local anesthesia is the frontline treatment of HSILs. Compared to conization approaches, LEEP removes less tissue, is quicker, requires less anesthesia, is less technically difficult, and generally less expensive than conization [34]. Conization is preferred when removing larger or deeper lesions [38]. For pathology analysis to inform on treatment options, cold-knife conization is the preferred option. Laser conization alters the excised tissue and LEEP introduces electrocautery artifacts that make histological evaluation challenging [39]. Rarely HSILS are addressed via hysterectomy, when other conditions make LEEP and conization impossible [40].

### 2.2 Areas for improvement with cervical cancer prevention

**Fig. 1:**
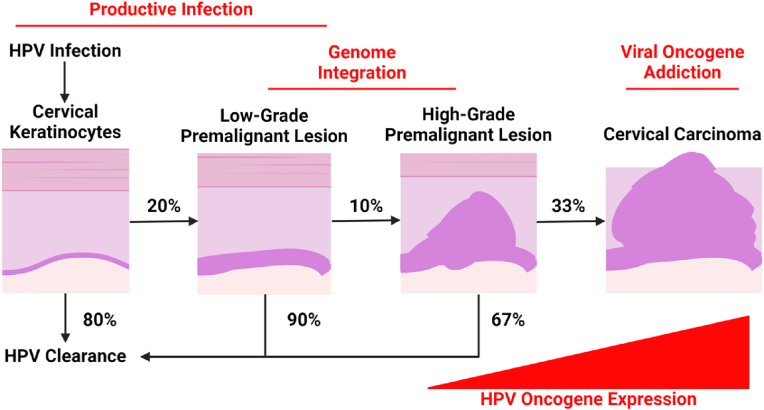
The Natural Progression of CaCx. This flowchart shows that most HPV-infected cells are cleared by the immune system without intervention. Only 20 % develop into low-grade premalignant lesions. Of these, 10 % progress to high-grade lesions, and only 33 % of high-grade lesions become carcinomas. The immune system eventually clears non-progressing lesions.

Although HPV vaccines are safe and effective, there are holes in their coverage. They do not afford protection against a group of less common high-risk strains (HPV 35, 39, 51, 56, and 59) that together are responsible for nearly 10 % of CaCx [41]. The nona-valent vaccine is only stable for 72 h outside of refrigeration, which limits its distribution in areas of the world with the greatest CaCx burden [42]. This contributes to about 10-fold lower rates of full HPV vaccination in females living in low-income countries [43]. Additionally, the HPV vaccine's relatively high cost can be prohibitive for Ministries of Health [[14], [15], [16]]. To address these issues, new vaccine candidates, formulations, and production methods are being developed. Vaccines that target the highly-conserved L2 capsid protein rather than the more variable L1 protein may provide a broader spectrum of immunity against high-risk strains [44]. Additionally, heat-stable [45] and *E. coli*-produced [46] variants of the vaccine seek to improve accessibility. Even in countries with well-developed infrastructure and where vaccination is accessible, vaccine hesitancy contributes to suboptimal vaccine uptake [[47], [48], [49]]. Unfortunately, these trends are likely to continue as people 18–34 years old have high levels of mistrust for vaccine safety [50]. There are limitations to secondary preventions as well. Existing HPV tests do not distinguish between integrated and circular HPV genomes, despite the former representing a greater risk of transformation [25]. Pap tests cannot triage HSILs based on their relative risk to progress to CaCx. This is notable as approximately two thirds of HSILs will regress without intervention [51]. More information on the natural progression of CaCx is included in Fig. 1. Similar limitations exist for other HSIL detection methods, so treatment is recommended for all HSILs [[52], [53], [54]]. Overtreatment results in unnecessary risks including infertility and an incompetent cervix, which can place a pregnancy at risk of premature labor and delivery [33,53].

## 3 Opportunities to improve cervical cancer prevention

While the limitations discussed are not newly discovered, technological advances may offer novel opportunities to address them. For example, improved sequencing technology may soon lead to the identification of biomarkers capable of distinguishing between HSILs that are and are not likely to progress to CaCx. Ideally, these biomarkers could be assessed in Pap test samples. However, existing efforts using qPCR and bulk RNA sequencing (RNAseq) have been unsuccessful [55,56]. This is likely because Pap test samples include a variety of cell types and bulk RNAseq averages expression across all cells in a sample. This obscures any predictive expression patterns that only existed in a subpopulation of HPV infected cells [57].

Single-cell RNAseq or scRNAseq of Pap test samples could overcome this limitation by identifying crucial cell subpopulations in patients, including immune and HSIL cells. scRNAseq works by “tagging” cDNA generated from mRNA in individual cells with short DNA barcodes that allow for the grouping of reads that originate from the same cell [58]. In addition to allowing the identification of subpopulations of cells based on differential gene expression, scRNAseq allows identification of HPV type. Further, our unpublished data have shown that scRNAseq can be performed without altering standard collection, storage, or processing of Pap samples. While scRNAseq analyses of Pap test samples have been implemented, more studies are needed to robustly access gene expression variations [[58], [59], [60]]. To identify predictive markers, a longitudinal scRNAseq study is needed. Better fixative agents are also improving the preservation of RNA and DNA in Pap test samples [61].

Identifying biomarkers by scRNAseq presents implementation problems. It is fiscally and computationally expensive, so it is a poorly matched approach for screening even in high resource settings. In-Cell PCR could address these barriers. It allows intracellular detection of RNA and DNA in specific cell populations and effectively genotype cells in mixed populations [62], allowing it to address the heterogeneity in Pap samples. The technical and resource requirements for In-Cell PCR are low, making it cost-effective and appropriate for screening. Thus, biomarkers identified through scRNAseq of Pap test could be used to direct the development of more affordable In-Cell PCR assays.

## 4 Efforts to improve cervical cancer chemotherapy

This section will begin with a review of the current best practices for chemotherapy in CaCx, including a discussion of the limitations and challenges of these approaches. We will next discuss ongoing and envisioned efforts to address these challenges.

### 4.1 Current standards of CaCx chemotherapy

Chemotherapy is typically used in advanced CaCx as an adjuvant to surgery [51]. It is also used for locally advanced CaCx. Cisplatin, the most widely-used chemotherapeutic for CaCx [63], is a platinum-based chemotherapy that can induce apoptosis by forming DNA adducts that result in DNA damage and cell cycle arrest [64]. These pathways are often dysregulated in CaCx cells, rendering them particularly vulnerable to cisplatin's effects [65,66]. Derivatives of cisplatin (e.g., carboplatin) have also been deployed with some success [[67], [68], [69]]. Cisplatin is often given combined with other treatments. The exact combination is determined by the severity of the disease and escalates with disease stage. For early-stage disease, surgery is common with or without lymph node surveillance. Whenever possible, fertility sparing surgeries are prioritized over hysterectomies. However, when early-stage tumors are inoperable, radiotherapy is employed. If nodes are positive, surgery is supplemented with radiotherapy (external beam radiation (EBRT) and brachytherapy) as well as cisplatin or other platinum-containing chemotherapy.

**Fig. 2:**
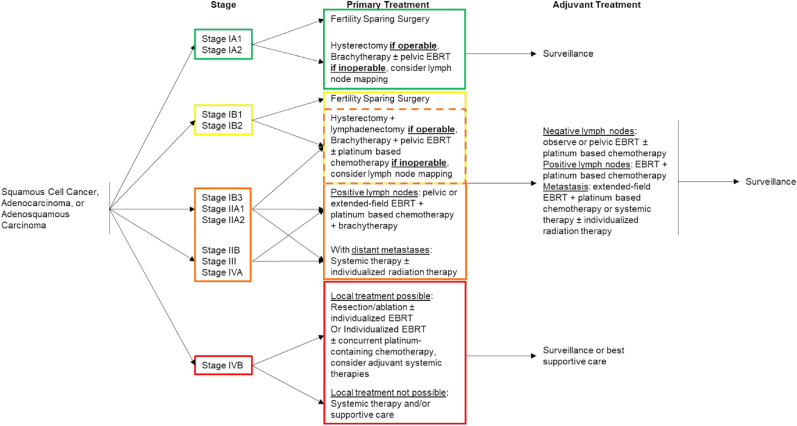
Flowchart of Treatment Options by Stage. Adapted from the NCCN cervical cancer guidelines. Systemic therapy refers to an array of different treatment options. Selection depends on the presence or absence of biomarkers. Surveillance refers to a range of surveillance methods. For a detailed layout of treatment options by stage consult the NCCA cervical cancer guidelines.

Advanced and recurring CaCx is treated with a combination of surgery, external beam radiation and cisplatin [70,71]. This combined approach reduces recurrence and increases overall and progression-free survival [70]. Concomitant chemo- and radio-therapy results in increased acute toxicity and has an unclear effect on long-term morbidities [[70], [71], [72]]. Palliative care of CaCx that is not considered curable has historically been cisplatin as a monotherapy. More recently, drug combinations consisting of cisplatin or carboplatin with paclitaxel (an inhibitor of microtubule disassembly, a key process in cell division [73]) or topotecan (an inhibitor of topoisomerase I, a key protein for DNA synthesis [74]) have been shown to better manage symptoms, improve quality of life, and extend overall survival [[75], [76], [77]]. The triple-combination of cisplatin, paclitaxel, and bevacizumab (an inhibitor of vascular endothelial growth factor A, a protein that promotes angiogenesis [78]) appears to offer further improvements [79]. More detailed recommendations for CaCx care and staging can be found in the National Comprehensive Cancer Network (NCCN) guidelines for CaCx and are summarized in Table 1 and Fig. 2.

**Table 1:** Stages and Sub-stages of CaCx and their Differentiation Criteria.

Stage	Location	Depth	Dimension	Effect on Normal Function	Metastasis
**I**	strictly confined to cervix				
IA			microscopic		
IA1		≤3 mm			
IA2		3 ≤ invasion ≤ 5 mm			
IB	limited to cervix uteri	no longer considered	>5 mm		
IB1			≤2 cm		
IB2			≤4 cm		
IB3			>4 cm		

**II**	invasion beyond uterus, but not to lower third of vagina or pelvic wall				
IIA	limited to the upper two-thirds of the vagina, no parametrial invasion				
IIA1			≤4 cm		
IIA2			>4 cm		
IIB	with parametrial invasion, but no involvement of pelvic wall				

**III**	extension into the lower third of vagina or to pelvic wall			causes hydronephrosis or non-functioning kidney and/or involves pelvic and/or paraaortic lymph nodes	
IIIA	extension into the lower third of vagina but not to pelvic wall				
IIIB	Extension to the pelvic wall			hydronephrosis or non-functioning kidney	
IIIC	Involvement of pelvic and/or paraaortic lymph nodes				micrometastases
IIIC1	spread to pelvic lymph node				pelvic lymph node metastasis only
IIIC2	spread to paraaortic lymph node				paraaortic lymph node metastasis

**IV**	spread beyond pelvis, involving the mucosa of bladder or rectum				yes
IVA	spread to adjacent organs				adjacent organs
IVB	spread to distant organs				distant

### 4.2 Immunotherapy

CaCx immunotherapy consists of therapeutic vaccines, immune checkpoint inhibitors (ICI) and adoptive T-cell therapy. Therapeutic vaccines have had some success in clinical trials of patients with preinvasive neoplasia, but have limited efficacy in invasive carcinoma [80]. Ongoing efforts have focused on enhancing their efficacy through combination with ICIs or other therapeutics. ICIs work by reactivating the immune response that becomes suppressed during tumorigenesis [81]. Their use is predicated on the presence of markers that predict their efficacy. The major mechanisms by which ICI re-activates the immune system include inhibiting the PD-1 surface protein on T-cells or the corresponding ligand PD-L1 on cancer cells. Pembrolizumab is an approved monoclonal antibody binding PD-1 and used as first- and second-line treatment in CaCx [82]. Similarly, nivolumab is recommended for use in PD-L1 positive tumors [83]. Other drugs in clinical trials target PD-1 (cemiplimab) [84], TGF-β (Bintrafusp alfa) [85] and CTLA-4 (ipilimumab) [86,87]. ICI monotherapy is currently considered to have unsatisfactory overall response rates [81], but many clinical trials for combination therapy are underway. The combination of platinum-based chemotherapy with pembrolizumab improved overall CaCx survival in a phase III clinical trial [88]. An extensive review of ICIs and CaCx can be found here [81]. Adoptive cell therapy (ACT) uses an infusion of tumor infiltrating lymphocytes (TILs) to inhibit tumor growth. TILs are extracted from surgically harvested tumors and expanded *ex vivo*. Patients receive a single TIL infusion after lymphodepletion. In an ongoing clinical trial, NCT03108495, ACT has shown positive results, but overall the approach requires further testing and development [89]. A detailed review of other ongoing ACT efforts for CaCx can be found here [90].

### 4.3 Areas for improvement with cervical cancer care

The radiotherapy and platinum-based drugs typically used to treat CaCx work by damaging DNA indiscriminately. They achieve some specificity by exploiting defects in DNA-repair and cell cycle regulation, common across cancers. While initially effective, acquired resistance is common [91]. Further, the indiscriminate nature of platinum-based compounds frequently leads to severe and dose-limiting side effects (e.g., nephrotoxicity, ototoxicity, nausea, vomiting [92]) with devastating impact on patient quality of life. These factors lead to an overall poor response to monotherapy in advanced and/or recurring disease [93,94]. While initial testing of immunotherapy approaches provides reason for optimism, it is unclear if they will work for all patients or if there will be unforeseen toxicities. Further, immunotherapy is currently laborious and expensive, making it a poor match for the areas of the world with the highest CaCx burden.

### 4.4 Opportunities to improve cervical cancer care

One way to reduce side effects from chemotherapy is to use drugs that more preferentially target cancer cells. This can be achieved by targeting pathways that are more often used by CaCx cells (e.g., cell proliferation or angiogenesis) or pathways altered specifically in these cells (DNA damage repair). Inhibitors of Wee1, a kinase that orchestrates mitotic entry and exit via CDK1 phosphorylation, have shown good efficacy in combination with radiation in patient-derived xenograft mouse models of CaCx [95,96]. In a phase I clinical trial this combination showed a good disease response but also increased morbidity [97]. Because tumors require angiogenesis to grow larger than 2 mm^3^ in size [98], inhibiting the angiogenic factor, vascular endothelial growth factor or VEGF, has been used to stunt CaCx tumor vascularization and improve survival [79]. In addition to the VEGF inhibitor (Bevacizumab) already approved for advanced and recurring CaCx treatment, there are ongoing clinical trials with inhibitors to VEGF receptors [99,100]. The approaches will likely continue through established mechanisms of drug development.

Because HPV oncogenes remain ubiquitously expressed in nearly all CaCx [101,102], there are efforts to improve CaCx care by targeting the HPV oncogenes using CRISPR/Cas9 [103] or small molecule inhibitors [104]. Because HPV E6 and E7 cause widespread changes to DNA repair mechanisms [[105], [106], [107]], it may be possible to target these viral proteins indirectly. Indeed, small molecule inhibitors have been developed to many of the repair pathways altered HPV oncogenes [[108], [109], [110], [111]]. It is possible that these inhibitors could be matched to specific HPV oncogene-induced changes in DNA repair, such that CaCx cells are specifically sensitized to genotoxic agents like cisplatin or radiation. However, this requires a detailed knowledge of which DNA repair pathways are differentially active in CaCx. For example, the activity of PARP1, an ADP-ribosylating enzyme involved in repair, is higher in CaCx, which motivated multiple promising concluded (NCT01281852, NCT737664) and ongoing (NCT03476798, NCT03644342) clinical trials using PARP1 in CaCx [[112], [113], [114], [115]]. The cellular response to DNA crosslinks (like those caused by cisplatin) is also frequently altered in CaCx. A deubiquitinating factor, USP1, that is required for tolerance and repair of these lesions is frequently overexpressed in CaCx [[116], [117], [118], [119]]. The cellular response to DNA crosslinks is further dysregulated by HPV oncogenes, which hinder the repair and tolerance of cross linked DNA [110,111,[120], [121], [122], [123], [124]]. These findings could be leveraged therapeutically as USP1 inhibitors are an emerging class chemotherapy currently being pursued for treatment of BRCA1/2 mutated tumors [[125], [126], [127]]. HPV oncogenes also dysregulate DNA double strand break (DSB) repair [109,128]. The culmination of this dysregulation is that CaCx are more likely to repair DSBs through a lesser used DNA repair pathway, known as alternative end joining (Alt-EJ) or microhomology mediated end joining. Alt-EJ is most commonly employed when other repair pathways stall [[129], [130], [131], [132], [133], [134]]. This comes at the cost of increased mutagenicity and chromosomal instability as Alt-EJ is more error-prone than more commonly used DSB repair mechanisms. Expression of POLθ, the rate limiting enzyme for Alt-EJ, is elevated in CaCx [[135], [136], [137], [138]]. These data suggest that POLθ inhibitors could have some utility as sensitizing agents. Several have been recently described (e.g., ART812) [[139], [140], [141]], two of which are being evaluated in ongoing clinical trials.

## Concluding statement

This review discusses recent technological advances that offer ways to address several long-standing barriers to CaCx prevention and care. We believe that we are rapidly approaching an era where Pap tests are more predictive and require less healthcare infrastructure. We also believe that chemo- and radio-sensitizing agents will reduce the harmful side effects of caring for CaCx.

## CRediT authorship contribution statement

**Grant Brooke:** Writing – review & editing, Writing – original draft, Visualization, Conceptualization. **Sebastian Wendel:** Writing – review & editing, Writing – original draft, Visualization, Conceptualization. **Abhineet Banerjee:** Writing – review & editing, Writing – original draft, Conceptualization. **Nicholas Wallace:** Writing – review & editing, Supervision, Project administration, Funding acquisition, Conceptualization.

## Declaration of competing interest

The authors declare that they have no known competing financial interests or personal relationships that could have appeared to influence the work reported in this paper.

## Data Availability

No data was used for the research described in the article.
